# Reduced disease in black abalone following mass mortality: phage therapy and natural selection

**DOI:** 10.3389/fmicb.2014.00078

**Published:** 2014-03-18

**Authors:** Carolyn S. Friedman, Nathan Wight, Lisa M. Crosson, Glenn R. VanBlaricom, Kevin D. Lafferty

**Affiliations:** ^1^School of Aquatic and Fishery Sciences, University of WashingtonSeattle, WA, USA; ^2^Washington Cooperative Fish and Wildlife Research Unit, U.S. Geological Survey, University of WashingtonSeattle, WA, USA; ^3^Western Ecological Research Center, U.S. Geological Survey, c/o Marine Science Institute, University of California at Santa BarbaraSanta Barbara, CA, USA

**Keywords:** abalone, *Haliotis*, withering syndrome, rickettsial, endangered, histology, selection, phage

## Abstract

Black abalone, *Haliotis cracherodii*, populations along the NE Pacific ocean have declined due to the rickettsial disease withering syndrome (WS). Natural recovery on San Nicolas Island (SNI) of Southern California suggested the development of resistance in island populations. Experimental challenges in one treatment demonstrated that progeny of disease-selected black abalone from SNI survived better than did those from naïve black abalone from Carmel Point in mainland coastal central California. Unexpectedly, the presence of a newly observed bacteriophage infecting the WS rickettsia (WS-RLO) had strong effects on the survival of infected abalone. Specifically, presence of phage-infected RLO (RLOv) reduced the host response to infection, RLO infection loads, and associated mortality. These data suggest that the black abalone: WS-RLO relationship is evolving through dual host mechanisms of resistance to RLO infection in the digestive gland via tolerance to infection in the primary target tissue (the post-esophagus) coupled with reduced pathogenicity of the WS-RLO by phage infection, which effectively reduces the infection load in the primary target tissue by half. Sea surface temperature patterns off southern California, associated with a recent hiatus in global-scale ocean warming, do not appear to be a sufficient explanation for survival patterns in SNI black abalone. These data highlight the potential for natural recovery of abalone populations over time and that further understanding of mechanisms governing host–parasite relationships will better enable us to manage declining populations.

## INTRODUCTION

The black abalone, *Haliotis cracherodii*, was once abundant along rocky shores of the NE Pacific (Haaker et al., 1986; Geiger, 1999). In some locations, such as the California islands, black abalone were often stacked 4–5 deep (Douros, 1987). Black abalone populations supported indigenous subsistence fisheries for at least nine millennia (e.g., Erlandson et al., 1996) and, more recently, supported a relatively stable commercial harvest (Karpov et al., 2000) until 1982 when a strong El Niño Southern Oscillation (ENSO) preceded catastrophic population declines (Dayton and Tegner, 1984). Near extirpation of black abalone was documented at many sites (up to 99% losses; Tissot, 1991, 1995; Haaker et al., 1992; VanBlaricom et al., 1993; Altstatt et al., 1996; Friedman et al., 2000; VanBlaricom et al., 2008; Neuman et al., 2010; Blaud, 2013). Due to precipitous declines, the black abalone became the second abalone species to be listed as “endangered” pursuant to the US Endangered Species Act (ESA) of 1973 as amended (16 US Code § § 1531-1543 et seq.) on January 14, 2009 (74 US Federal Register 1937). Concern over the ability of depleted populations of black abalone to recover following mass mortality was heightened by the fact that recruits have been shown to largely originate from local stocks (Hamm and Burton, 2000; Chambers et al., 2005, 2006).

The post-ENSO mass mortalities resulted from an infectious disease called withering syndrome (WS). The etiological agent of WS is an intracytoplasmic rickettsia-like organism (WS-RLO), “*Candidatus* Xenohaliotis californiensis,” which infects abalone gastrointestinal epithelia (Friedman et al., 2000). WS has also impacted populations of green, *H. fulgens*, and pink abalone, *H. corrugata*, in Mexico (del Álvarez et al., 2002) and contributes to seasonal losses of cultured red abalone, *H. rufescens*, in California, particularly during ENSO conditions (Moore et al., 2000; Braid et al., 2005; Vilchis et al., 2005). The endangered white abalone (*H. sorenseni*) is known to be vulnerable to mortality caused by WS in captivity, but population-scale effects in natural habitats are unknown (Friedman et al., 2007; Crosson et al., 2014). WS is now endemic in California and is an impediment to recovery of affected abalone species (Moore et al., 2002; Friedman and Finley, 2003; Friedman et al., 2007).

Infections with the WS-RLO begin in the posterior esophagus (PE) epithelium and cause morphological changes (metaplasia) in the digestive gland (DG) of the abalone host that enable the bacterium to infect this organ (a secondary target tissue for the WS-RLO; Friedman et al., 2000) and disrupt its function leading to catabolism of the foot muscle to obtain energy and, finally, death (Friedman et al., 2000; Braid et al., 2005). Clinical disease occurs in RLO-infected animals during periods of elevated seawater temperatures (e.g., ≥18°C; Moore et al., 2000; Friedman et al., 2002; Braid et al., 2005; Vilchis et al., 2005). However, a recent study comparing the influence of ENSO with La Niña and ambient (“normal”) temperature conditions illustrated that the thermal threshold for WS may be lower than 18°C (Moore et al., 2011). Thus the severity and spread of WS is linked to climatic variation (temperature). In black abalone, temperature variation was shown to increase susceptibility to infection, while high mean temperatures increase mortality rates of infected individuals (Ben-Horin et al., 2013).

Some black abalone survived the post-ENSO mass mortalities, leading to the speculation that remaining individuals might be somehow less affected by infection (Lafferty and Kuris, 1993). With this possibility in mind, the black abalone fishery was closed in 1993 to protect whatever genetic variation remained [California Department of Fish and Game (CDFG), 1993]. From 95 to 99% of black abalone in nine permanent intertidal study sites at San Nicolas Island (SNI, which is located off Southern California; VanBlaricom et al., 1993) died within 10 years of WS’s emergence at the island in spring 1992 (VanBlaricom et al., 1993). All study sites experienced mass mortalities, but beginning in 1996 it was apparent that survival rate was higher at study site 8 than at other SNI study sites (Chambers et al., 2005, 2006; Crosson et al., 2014). Subsequently, recruitment of black abalone was observed on SNI beginning in 2002 and was especially marked at site 8 (Chambers et al., 2005, 2006). A shift in climate trend to a “hiatus” in warming over the past decade (e.g., Easterling and Wehner, 2009; Kosaka and Xie, 2013) may have contributed to observed recruitment but did not appear to fully explain this change. In particular, an ENSO event that occurred in 2004–2005 in Southern California (Ben-Horin et al., 2013) and the fact that recruitment on SNI has occurred primarily in one locale suggest that temperature alone is not driving recovery of black abalone on SNI (Crosson et al., 2014). These observations led to the hypothesis that the young abalone on SNI might be progeny of parents with heritable traits that had helped them survive the past mass mortality events.

To test this hypothesis, we undertook studies to characterize differential susceptibility or resistance to WS of newly emergent recruits among populations of black abalone using controlled laboratory challenges. In the absence of acquired immunity, there are three ways that hosts might evolve in response to a pathogen: decreased susceptibility (inability to become infected), increased resistance (control of the pathogen once infected), or increased tolerance (lack of disease despite infection; Boots and Bowers, 1999). We formulated specific predictions for these three hypotheses. If past exposure had selected for reduced susceptibility, we predicted that abalone from SNI (disease-selected abalone) would be less likely to become infected when exposed to the WS-RLO compared with abalone from Carmel (naïve abalone). If past exposure had selected for resistance, we predicted that selected abalone would have lower-intensity infections in one or both target organs (PE and DG) after a successful exposure compared with naïve abalone. If past exposure had selected for tolerance, we expected selected abalone would show a weaker relationship between RLO intensity (specifically the intensity of RLO in the DG) and pathology associated with mortality (metaplasia and/or foot condition) compared to naïve abalone.

During the course of our studies, a phage hyperparasite was observed in black abalone infected with the WS-RLO (phage-infected WS-RLO is named RLOv) and was hypothesized to reduce the intra-host replication of the parasite (Friedman and Crosson, 2012). Therefore, we also predicted that presence of the RLOv would reduce disease in all exposed abalone.

## MATERIALS AND METHODS

### GENERAL METHODS

#### Animals

Black abalone ranging in size (defined as the maximum length of the elliptical shell) from 25 to 67 mm were collected from three sites at two study areas to reduce impacts from collections on the populations prior to ESA listing. Abalone were collected from SNI sites 6 (33.215°N, 119.475°W; *n* = 10), 7 (33.219°N, 119.497°W; *n* = 16) and 8 (33.231°N, 119.534°W; *n* = 85) on February 26, 2006 (mean size ± SD = 36.23 ± 8.45 mm; and from the greater Carmel area in central California (Carmel) on February 23–24, 2006 (mean size = 46.22 ± 11.02 mm). Carmel animals were collected from three sites including Granite Canyon (36.436°N, 121.920°W; *n* = 37), Carmel Point (36.544°N, 121.933°W; *n* = 37), and Soberanes Point (36.448°N, 121.929°W; *n* = 30). We received 30 RLO infected red abalone (size range 75–100 mm) from The Abalone Farm, Inc. (35.438°N, 120.894°W; Cayucos, CA, USA) to serve as donor animals to infect black abalone in Trial 1 and used black abalone (*n* = 9 that measured 40–60 mm) from SNI (site 8) as donor animals in Trial 2. In addition, we received 60 red abalone (mean size = ~30–40 mm) from The Abalone Farm to serve as controls for tank independence in our trials. Following collection in the field all abalone were transported overnight on ice to the School of Aquatic and Fishery Sciences-University of Washington Pathogen Quarantine Facility (UWPQF) and placed in separate recirculating seawater systems at 14°C for an 8-week acclimation period prior to experimental manipulations. Seawater (~30 psu) was collected from Elliot Bay, Puget Sound, WA, USA and transported to the UWPQF where it was added to each system and maintained at the desired temperature via a heat pump (Delta Star, AquaLogic, Inc., San Diego, CA, USA) and was purified via filtration (25 μm) and ultra violet irradiation followed by an activated charcoal filter. Selected seawater parameters (ammonia, nitrite, and pH) were measured 2× per week prior to weekly partial (~30%) water changes. Temperature and animal mortality were monitored daily. Animals were fed the algae *Nereocystis luetkeana* (Phaeophyta, Lessoniaceae), *Palmaria mollis* (Rhodophyta, Palmariaceae), and/or *Chondracanthus exasperatus* (Rhodophyta, Gigartinaceae) 2–3× per week. Periodically, feces were collected from each system for assessment of WS-RLO presence via quantitative polymerase chain reaction (qPCR; Friedman et al., 2014). Feces (1–2 ml) were aspirated from each system and the DNA was immediately extracted as outlined below. All effluent from this facility was chlorinated at 10 ppm for 24 h prior to release in the Seattle domestic sewer system as per requirement by the Washington Department of Fish and Wildlife.

#### Administration and analysis of oxytetracycline

The red abalone (~30–40 mm group) and all black abalone were medicated with oxytetracycline (OTC) at a dose of ~90 mg/kg weight for 3 days according to the methods of Friedman et al. (2007) in order to allow the abalone to purge themselves of RLO infections (Friedman et al., 2003, 2007; Rosenblum et al., 2008). Abalone were maintained in recirculating seawater systems held at 14–15°C for 4 months to allow depletion of OTC to <50 ppm, a level shown to allow re-infection by the WS-RLO (Friedman et al., 2007; Rosenblum et al., 2008). OTC levels in the DG were analyzed according to Association of Official Analytical Chemists [AOAC] (1990) as modified by Friedman et al. (2003, 2007).

#### Histology

A standard 2–3 mm cross section was excised from each animal sampled to include PE, DG, and foot muscle from all moribund abalone and those sampled at specific time points. Excised tissues were preserved in Davidson’s solution (Shaw and Battle, 1957) for 24 h and stored in 70% ethanol until being processed by routine paraffin histology. Deparaffinized 5 μm sections were stained with hematoxylin and eosin (Luna, 1968) and viewed by light microscopy. One to three morphologically distinct RLOs were observed in histological sections and included the WS-RLO, a phage-infected WS-RLO variant (RLOv) and a previously undescribed RLO that is stippled in appearance (ST-RLO; Friedman et al., 1997, 2000; Friedman and Crosson, 2012). The intensities of infections by each RLO type were individually scored according to the following 0–3 histology scale estimating the number of rickettsial colonies per 20× field of view: (0) no infection, (1) 1–10, (2) 11–100, and (3) >100 (Friedman et al., 2002). Tissue changes (metaplasia or foot atrophy) were also scored on the 0–3 scale of Friedman et al. (2002) in which 0 represented normal tissue, 1 indicated <10% change, 2 indicated 11–25% change, and 3 indicated >25% change.

#### DNA extractions

As the PE is the primary target organ for infection by the WS bacterium (Friedman et al., 2000), this tissue was selected for optimum RLO detection by histology and qPCR, the main diagnostic tools employed in these studies. PE tissue for histology was removed just posterior to the right kidney–DG junction and the next posterior section that contained PE and DG was excised for qPCR. These two sections represent the portion of the gastrointestinal tract where RLO infections are most prevalent (Friedman et al., 2000, 2002, 2007). DNA isolation from PE and DG tissues was performed with a QiaAmp DNA Stool Mini Kit (Qiagen Inc., Valencia, CA, USA) according to the manufacturers’ instructions as modified by Friedman et al. (2007). DNA isolation from feces was performed in the same manner except that initial homogenization was not necessary. All extracted DNA was stored at –20°C until further analysis.

#### Quantitative PCR

Quantification of WS-RLO DNA was accomplished using the qPCR assay of Friedman et al. (2014). qPCR reactions were conducted using 12.5 μl 2× Immomix (Bioline USA Inc., Taunton, MA, USA), 320 nM of each primer, 200 nM of probe (Biosearch Technologies, Inc., Novato, CA, USA), 0.6 mg/μl BSA, 2 μl of DNA template, and sterile water to bring the final volume to 25 μl per reaction. Thermal cycling conditions included an initial denaturation step of 95°C for 10 min, followed by 41 cycles of 95°C for 15 s, and 60°C for 30 s. Each sample was run in triplicate along with a plasmid-based standard curve of known WS-RLO copy numbers and a negative control. The fluorescence threshold was set at 400 dR for quantification cycle (Cq) determination to allow precise comparison of amplification among reactions. WS-RLO copy numbers were determined for each abalone DNA sample via regression analysis of the standard curve. Gene copy numbers were calculated per gram of sample for tissues and per nanogram of genomic DNA for fecal sample.

### EXPERIMENTAL METHODS

#### Trial 1: Multiple RLO infections: WS-RLO, RLOv, and ST-RLO

**FIGURE 1:**
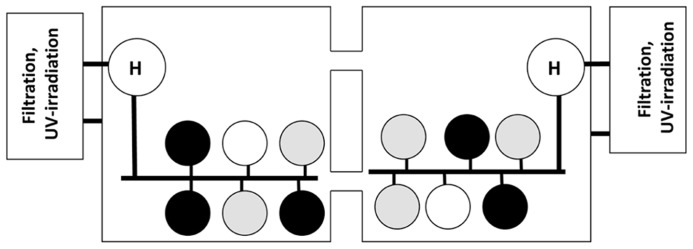
**Exposure system used in challenge Trials 1 and 2.** Abalone from SNI (black circles) and GC (gray circles) were held in replicate tanks (*n* = 5 in Trial 1 and 2 in Trial 2). The white circles represent tank of uninfected red abalone to test for tank independence in the recirculation system. Seawater flowed from the head tanks (H) holding either infected or uninfected abalone into the exposure tanks. Subsequently seawater flowed into the square sumps and through a series to filters, UV-sterilization and heating systems prior to being returned to the head tank.

After depletion of OTC, abalone from both collection sites (Carmel and SNI) were equally divided among 10 plastic aquaria [12 in × 13.5 in (D × H); Consolidated Plastics, Twinsburg, OH, USA] so that each contained eight abalone and were, in turn, distributed among four 400-l recirculating seawater systems. Two systems were designated as control (no RLO exposure) and two as experimental (RLO exposure) treatments. Each system held two or three tanks from the two black abalone collection sites (SNI and Carmel) for a total of five tanks of black abalone from each population. Each system included a head tank from which water flowed into tanks (**Figure 1**). Abalone were acclimated to the target seawater temperature of 18°C over a 1-week period and maintained at this temperature throughout the study. Seawater systems were maintained and abalone were fed as described above.

One week after reaching 18°C, an equal biomass of red abalone (611 g) was added to each of the four head tanks. RLO-infected red abalone were placed in the head tanks of the experimental systems providing an equivalent dose of the pathogen to each tank, while those free of RLO infection were added to the control systems. After 60 days, red abalone were removed from the head tanks and a sixth tank containing 15 uninfected red abalone was added to each of the four systems (system controls for tank independence).

Each system was checked daily for the presence of moribund (lethargic and weakly attached) animals, which were promptly removed. Selected tissues (PE, DG, and foot muscle) were excised for qPCR and histological analyses. After 162 days, one abalone was removed from each tank and sampled as above. Upon termination of the study, all remaining experimental abalone and two control abalone per tank were sampled as above.

Due to the paucity of abalone available for this study, to better understand host–parasite dynamics, fecal samples were periodically removed from tanks 24 h after feeding and processed for qPCR according to Friedman et al. (2014).

#### Trial 2: Dual RLO infections (WS-RLO and ST-RLO)

In an effort to compare relative susceptibility of the Carmel and SNI populations without the newly observed RLOv, we repeated the above study using the remaining control abalone and donor abalone from SNI, where the RLOv has not been observed (Friedman and Crosson, 2012). The trial was conducted in the same manner as above with the exception that duplicate tanks holding five animals each were used from each population under the same control and experimental conditions as outlined above. No abalone were sampled during the study except for moribund or dead individuals. All remaining animals were sacrificed and sampled as above.

#### Trial 3: Single RLO infections (WS-RLO)

We re-analyzed data from Friedman et al. (2002) in which black abalone from Año Nuevo Island (37.1086°N, 122.3378°W) were exposed to the WS-RLO via cohabitation under the same temperature conditions as in Trials 1 and 2 (18°C) but were held in flowing seawater in lieu of recirculating seawater systems. Abalone used in Trial 3 were larger (mean size = >100 mm) than those used in Trials 1 and 2 (mean size = 49 and 57 mm, respectively). Moribund and surviving abalone were sampled for histology as described above. New analyses of histology and survival data were conducted and are outlined below.

### SEA SURFACE TEMPERATURE DATA

Our primary source for sea surface temperature (SST) data at SNI was waverider data buoy 46219 (33.221°N, 119.882°W), moored 32.4 km west of SNI site 8 and operated by the US National Oceanic and Atmospheric Administration (NOAA). Buoy 46219 is also identified as buoy 067 of the Coastal Data Information Program, Scripps Institution of Oceanography, La Jolla, CA, USA. Between September 2004 and December 2012 a total of 42,677 publically available SST measurements were logged hourly, distributed across 46 different months at the buoy site. To verify that data from the buoy site represented SST conditions at SNI, we compared SST data collected in 2007 in the intertidal zone at SNI site 8 with data from the buoy. SNI data were collected between February 1 and December 31 using “TidbiT^®^” SST loggers (Onset^®^ Computer Corporation, Bourne, MA, USA) cemented to rocky substrata. The data sets included 106 records of paired (within 1 h of one another) SST measurements from the two locations that were logged within 1 h of high tide at SNI. Times of high tides were determined with Nobeltec^®^ Tides and Currents^TM^ software, version 3.5 (Jeppesen Marine, Portland, OR, USA). The requirement for observations at high tide ensured that the data loggers at SNI were fully immersed when SST records were obtained.

### STATISTICAL ANALYSES

Histology was used as the primary determinant of infection status. Abalone that were not exposed, but showed signs of infection (*n* = 2 in Trial 2), were discarded from analyses because these animals were most likely contaminated with RLO during the end of the trials. We did not include data from animals that were sampled for histology midway through the trials, but rather used data only from abalone that died or survived until the end of the trials. Our measure of phage infection (RLOv) was either present or absent or ranked density in histological preparations, depending on the analysis. Histological scores for foot condition (atrophy) and RLO density were assumed to be continuous variables on a log scale. Mortality rates of control abalone differed among the trials and this variation in background mortality was controlled for in analyses by nesting the effect of infection within trial. WS-RLO gene copies based on qPCR analyses were included as an additional estimate and acted as a proxy for RLO load (Friedman et al., 2014). To simplify the analyses, we pooled abalone across trials into two groups based on exposure history of disease-selected and naïve. Abalone from SNI were progeny of abalone selected for disease resistance (“selected”), while those from Carmel, where losses due to WS have not been observed (Miner et al., 2006) were considered “naïve.” Our survivorship models considered infected (vs uninfected) nested within trial, the interaction between infection status and exposure history, and the interaction between infection status and presence of phage. Here, the interaction terms were the main predictions of our hypotheses, i.e., that the mortality rate of infected abalone relative to control abalone is affected by phage or exposure history to RLOs. We used a proportional hazards model to determine the effect of exposure history and RLOv infection on the relative mortality rates of experimental abalone (for convenience, plots were made with parametric survivorship analysis using a Weibull distribution – results were consistent between the two approaches).

We used logistic stepwise regression to consider a chain of causal mechanisms leading to abalone mortality and pathological indices. For all models, we nested the main effect within trial. Least squares regression was used for all tests except for a nominal regression to contrast mortalities versus survivors. We assumed that all indices were continuous variables due to the presence of several fractional values. Treating these as ordinal variables did not alter the nature of the results. For the linear models, the normality of the residuals was inspected using normal quantile plots to insure that the assumptions of the test were met. All statistical analyses were run using JMP 10.0 (SAS, Cary, NC, USA).

We calculated Pearson’s *r* value to assess the strength of the correlation in SST data between NOAA data buoy 46219 and SNI site 8, using paired data consistent with selection criteria as described above.

## RESULTS

### SURVIVAL

**FIGURE 2:**
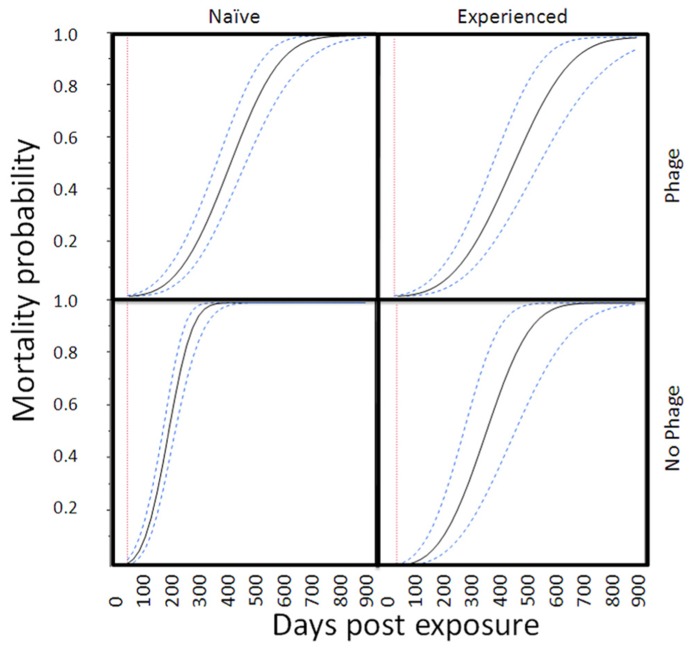
**Mortality curves for infected abalone in four categories.** The curves (mean values and 95% confidence intervals) are based on a parametric survival fit analysis with Weibull function. For figure simplification, we did not nest infection within trial. This model was consistent with statistics reported from the proportional hazards model.

**FIGURE 3:**
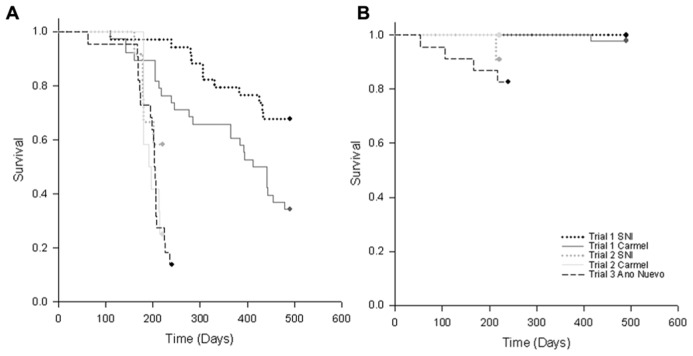
**Kaplan–Meier survivorship curves for **(A)** RLO-exposed and **(B)** control (unexposed) black abalone from Trials 1–3 in which abalone from Carmel and San Nicholas Island (SNI) were exposed to three RLOs (WS-RLO, ST-RLO, and RLOv) in Trial 1, two RLOs (WS-RLO and ST-RLO) in Trial 2, or one RLO (WS-RLO) in Trial 3.** Survival trends for abalone from the three collection sites are represented by dotted lines (SNI), solid lines (Carmel) and dashed lines (Año Nuevo Island).

In the proportional hazards survivorship analysis (78 deaths, 69 survivors), infected abalone had significantly higher mortality rates than control abalone (infection nested in site, χ^2^ = 29.4, df = 3, *P* < 0.0001). The relative mortality rate of phage-infected abalone was significantly lower than abalone without phage (interaction between infection and presence of phage, χ^2^ = 19.0, df = 1, *P* < 0.0001). There was a near-significant (two-tailed) effect of exposure history (interaction between infection and history of exposure, χ^2^ = 19.0, df = 1, *P* = 0.066), with naïve Carmel abalone suffering slightly higher mortality rates than abalone from disease-selected SNI populations, particularly in Trial 1. To illustrate the magnitude of these effects, **Figure 2** shows mortality curves for four categories of infected abalone. Here, the projected effect of phage (RLOv) was to extend the mean (with 95% confidence) time until 50% mortality in infected abalone from 189 (169–211) days (naïve) or 211 (175–251) (selected) days to 399 (352–450) days (naïve) or 443 (386–514) days (selected; **Figure 3**).

### HISTOLOGY

Abalone mortality significantly increased with pedal atrophy (withered foot) index (nested within trial, χ^2^ = 65.4, df = 3, *n* = 136), *P* < 0.0001). Pedal atrophy index significantly increased with DG-RLO index (nested within trial, *F*-ratio = 23.8, df = 3, *n* = 133, *P* < 0.0001). DG-RLO index significantly increased with metaplasia index (nested within trial, *F*-ratio = 67.6, df = 3, *n* = 143, *P* < 0.0001). Metaplasia index significantly increased with PE-RLO index (nested within trial, *F*-ratio = 67.6, df = 3, *n* = 143, *P* < 0.0001). PE-RLO index in infected abalone was not affected by exposure history (nested within trial, *F*-ratio = 0.03, df = 2, *n* = 93, *P* = 0.97). However, in Trial 1, more than half of the PE-RLOs were RLOv (phage-infected WS-RLO), preventing them from contributing to metaplasia and thereby reducing DG-RLO loads as evidenced by reduced DG-RLO indices in these animals (**Table 1**).

**Table 1:** Histology trends among groups sampled as mortalities relative to those that survived within each trial.

Trial	Site	Mortality or survivor	No.	Mean foot	Mean metaplasia	PE-RLO	PE-ST	PE-RLOv	DG-RLO	DG-ST	DG-RLOv	Mean total PE-RLOs	Mean total DG-RLOs
1	SNI^1^	M	12	**1.6**	*1.2*	1.5	0.6	1.8	*0.9*	0.4	0.8	2.0	1.1
	SNI	S	10	**0.1**	*0.3*	1.7	0.4	1.9	*0.4*	0.1	0.6	2.1	0.6
	Carmel	M	24	**2.0**	1.5	1.7	0.8	1.7	1.3	0.8	*1.0*	2.3	1.6
	Carmel	S	10	**0.5**	1.3	1.7	0.5	1.9	1.5	0.4	*1.5*	2.2	1.7
2	SNI	M	5	**2.1**	2.0	2.6	0.6	0.0	1.9	0.0	0.0	2.6	1.9
	SNI	S	4	**0.6**	1.7	2.3	0.6	0.0	1.5	0.5	0.0	2.3	1.7
	Carmel	M	10	2.7	2.3	2.3	1.8	0.0	**2.5**	0.6	0.0	2.4	**2.5**
	Carmel	S	2	2.1	1.7	1.3	1.3	0.0	**1.5**	1.3	0.0	1.8	**1.7**
3	Año^2^	M	21	1.5	*1.0*	2.3	0.0	0.0	1.5	0.0	0.0	2.3	1.5
	Año	S	3	2.0	*2.0*	2.7	0.0	0.0	2.3	0.0	0.0	2.7	2.3
1, 2, 3	Controls	S	39	0.1	0.1	0	0	0	0	0	0	0	0

*RLO infection intensities were scored by estimating the number of rickettsial colonies per 20× field of view: (0) no infection, (1) 1–10, (2) 11–100, and (3) >100 (Friedman et al., 2002). Tissue changes (metaplasia or foot atrophy) were similarly scored: 0 represented normal tissue, 1 indicated <10% change, 2 indicated 11–25% change, and 3 indicated >25% change. Bold indicates significant differences in measured response of exposed abalone between sampling periods (mortality or survivor) within a collection site. Italics indicate nearly significant differences between mortalities and survivors within a collection site (*P* = 0.052–0.069). Underlined data indicate significant differences in measured response between abalone with different exposure histories (collection site) and whether they were animals that died (mortality, M) or survived (S) to be sampled. ^1^San Nicolas Island. ^2^Año Nuevo Island.*

**FIGURE 4:**
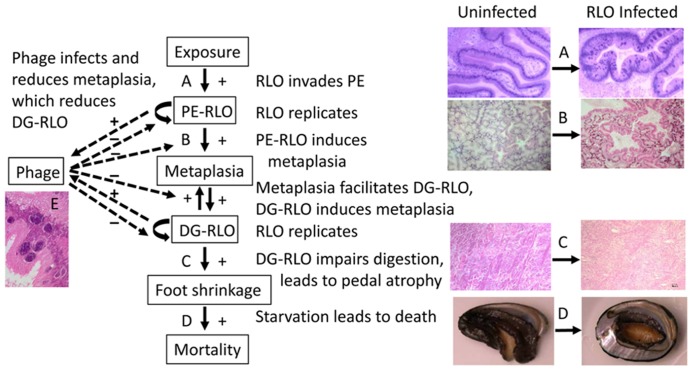
**Flow chart illustrating the relationship among abalone host, WS-RLO parasite, and phage hyperparasite of WS-RLO.** Arrows indicate the flow of the effect with an increase in the response denoted with a “+” and a reduction in the response shown by a “-”. WS-RLO exposure results in infection of the posterior esophagus (PE) **(A)**, which leads to metaplasia in the digestive gland (DG) **(B)**. Metaplasia, in turn, provides more target tissue for the WS-RLO to invade and infect this organ, which together result in dysfunction of the DG and catabolism of the pedal muscle as an energy source **(C)**. Visible atrophy of the pedal muscle becomes apparent in the withered and lethargic abalone at the end-stage of disease **(D)**. Phage infection of the WS-RLO (RLOv) reduces the amount of active WS-RLO, thereby reducing metaplasia and DG-RLO, which in turn reduces pedal atrophy and mortality **(E)**. Magnifications: **(A)** 100×, **(B,C)** 40×, **(D)** 1×, **(E)** 400×.

Given that exposure history did not influence infection potential based on similar PE-RLO between groups, we conducted a specific test to see if pathological changes due to infection varied between animals that survived and those that died in the three trials. We also examined if exposure history affected other aspects of pathology. Overall, animals that survived had lower levels of metaplasia (index of metaplasia = 0.56) than those that died (index = 1.37; *F*-ratio = 5.5, df = 7, *n* = 144, *P* < 0.01). Within each trial, metaplasia indices of survivors were lower than those that died, with index values of 0.39 and 1.35, respectively, in Trial 1 (*F*-ratio = 6.9, df = 3, *n* = 75, *P* < 0.001), 1.46 and 2.21, respectively, in Trial 2 (*F*-ratio = 1.98, df = 3, *n* = 27, *P* = 0.052), and 0.27 and 0.79, respectively, in Trial 3 (*F*-ratio = 4.5, df = 1, *n* = 40, *P* < 0.05). Presence of the phage (RLOv) reduced both metaplasia (index = 1.12 with phage and 1.68 without phage, *F*-ratio = 4.41, df = 1, *n* = 95, *P* = 0.039) and DG-RLO load (index = 1.38 with phage vs 1.90 without phage, *F*-ratio = 8.99, df = 1, *n* = 95, *P* = 0.0035). The effect of exposure history on infected animals was demonstrated in Trial 1 by the observation of reduced metaplasia upon infection in selected (index = 0.81) relative to naïve animals (index = 1.54; *F*-ratio = 5.30, df = 1, *n* = 54, *P* < 0.05). However, mean metaplasia index was similar between naïve (2.23) and selected (2.00) animals in Trial 2 (*F*-ratio = 0.307, df = 1, *n* = 21, *P* = 0.59; **Table 1**; **Figure 4**). Overall, exposure history affected metaplasia but the effect was not consistent across trials, suggesting, perhaps, that not all the exposed abalone had been under selection for increased resistance at SNI.

### qPCR

Naïve infected abalone from Carmel contained ~ 2.5 times more copies of the WS-RLO rDNA gene than did those from disease-selected WS-RLO-infected abalone from SNI [3.82 × 10^4^ (SE = 6.62 × 10^3^) vs 1.47 × 10^4^ (SE = 8.30 × 10^3^) copies per milligram tissue, respectively; *F*-ratio = 4.90, df = 1, *n* = 71, *P* = 0.03]. Abalone that died also contained a mean of 50% more copies of the WS-RLO DNA gene than did survivors. However, these differences were not significantly different from one another [4.28 × 10^4^ (SE = 6.85 × 10^3^) vs 1.28 × 10^4^ (SE = 8. × 10^3^) copies per milligram tissue, respectively; *F*-ratio = 2.10, df = 3, *n* = 71, *P* = 0.22]. Although infected Carmel abalone excreted 12 times more copies of RLO DNA per nanogram fecal DNA than did infected abalone from SNI [1.9 × 10^4^ (SE = 9.73 × 10^3^) vs 0.16 × 10^4^ (SE = 9.73 × 10^3^) copies per nanogram genomic DNA], these differences were not significant (*F*-ratio = 1.60, df = 1, *n* = 17, *P* = 0.22).

### SEA SURFACE TEMPERATURE

We found that 17.7% of SST records from NOAA buoy 46219 were ≥17°C and 5.3% ≥18°C (**Table 2**). During the period of record many events of ≥4 h duration occurred in which 17.0 í SST < 18.0°C, and with SST > 18.0°C (**Table 2**). The correlation between paired SST records from the buoy and from SST loggers at SNI site 8 was significant (Pearson’s *r* = 0.71, df = 104, *P* < 0.001).

**Table 2:** Frequency and duration of occurrence of sea surface temperatures (SSTs) sufficiently high to increase the risk of black abalone to effects of withering syndrome at San Nicolas Island (SNI), California USA.

	SST ≥ 17°C	SST ≥ 18°C	17 ≤ SST < 18°C	SST ≥ 18°C
Frequency (*n* = 42,677 hourly observations)	7,559 (17.7%)	2,253 (5.3%)	–	–
Number of episodes	–	–	200	73
Mean duration (range)	–	–	14.1 h (0.5–718 h)	15.9 h (0.5–257 h)

*Data are publically available and were collected hourly by NOAA waverider buoy 46219, located 32.4 km west of black abalone study site 8 at SNI. Data collection was intermittent from September 2004 through December 2012. Minimum episode duration was set at 4 h. Data from the buoy were significantly correlated with temporally paired SST data obtained during high tide in black abalone habitat at site 8 in 2007.*

## DISCUSSION

Our findings suggest that the increased survivorship seen at SNI was due to reduced metaplasia and WS-RLO loads in the DG. While we found some evidence for our hypothesis of the evolution of resistance in black abalone following mass mortality, the significance of these results varied among trials. It is possible that selection for resistance varied among the samples we used in our experiments (which were taken at different times). Furthermore, sample sizes were small in Trial 2, which combined with a shorter trial duration may have influenced our ability to detect differences in measured metrics between groups. Trial 2 (lacking phage) was terminated after day 85 (when no abalone deaths had occurred for 2 weeks). Although the slopes of the mortality curves for exposed animals in Trial 2 were similar, ~2× more SNI abalone survived than those from Carmel (**Figure 3A**). Thus, had the study continued longer, we might have seen differences in survival between exposure history arise as seen in trail one. Despite inferential constraints imposed by sample size limitation, differences in mortality of naïve relative to disease-selected abalone when exposed to the WS-RLO were consistent with effects observed in Trial 1.

The ability for marine mollusks to evolve disease resistance has been investigated in a number of species with varying results. A viral disease of abalone (abalone viral ganglioneuritis, AVG) in Australia caused high mortality along the coast of Victoria in 2005–2006 and surviving abalone tested 5–6 years later remained susceptible to the virus suggesting a lack of evolved resistance (Crane et al., 2013). The ephemeral nature of AVG and possibly insufficient time for significant recruitment post-selection may explain the apparent lack of developed resistance to this disease. The development of disease resistance has been investigated in oysters infected with protistan and viral pathogens. After four generations of selection beginning with American oyster *Crassostrea virginica* broodstock that had survived disease pressure from two protistan pathogens for 2–5 years, cumulative survival more than doubled when exposed to the protists (Calvo et al., 2003) illustrating the development of resistance over time. Resistance in populations has also been observed in Pacific oysters *Crassostrea gigas* after several generations of selection to a herpes virus (ostreid herpesvirus-1); heritability of traits was also high (e.g., h^2^ = 0.61–0.95; Dégremont et al., 2010). These examples illustrate a genetic basis for resistance in marine mollusks and that rates of selection for resistance vary among species. The black abalone from SNI were likely first or second generation progeny of those that survived the initial losses of ≥95% due to WS given their observation 10 years after the first observation of WS on SNI in 1992 and ~5–6 years after ~90% mortality (VanBlaricom et al., 1993; Crosson et al., 2014). It is possible that abalone have limited ability to evolve resistance to novel pathogens like AVG and WS-RLO; however our data suggest that over time enhanced resistance to the WS-RLO is likely to increase in populations experiencing disease pressure.

Given the known association of WS-induced mortality rates with warm-water events such as ENSO episodes, it is plausible that the recently observed global-scale hiatus in sea surface warming (e.g., Meehl et al., 2011; Kosaka and Xie, 2013) may have mitigated detrimental effects of WS on black abalone populations in southern California. However, we are skeptical that ocean temperature trends alone can explain observed positive trends in black abalone population densities at SNI and elsewhere in the southern California Islands for two reasons. First, our evaluation of relevant SST data indicated that periods of SST high enough to enhance vulnerability of black abalone to WS at SNI were frequent and often of substantial duration (continuous for up to 30 days), despite the hiatus in ocean warming. Second, we would have expected a regional climate signal to have led to signs of incipient population-scale recovery at more than just a small number of sites.

Our finding that the phage effectively eliminates the ability of infected WS-RLOs to cause disease was unexpected. Seasonal losses due to WS in California farms have lessened over the past several years since the phage was observed in farmed animals suggesting that natural phage-therapy operates under farm conditions (R. Fields, personal communication), and this effect may also apply to the field. However, whether or not phage commonly infects wild abalone remains to be seen.

Viroplankton (especially bacteriophages) are the most abundant biological elements in the marine environment and play key roles in driving host population dynamics (see review by Suttle, 2005). The observation of a phage hyperparasite in the WS-RLO is therefore not surprising. A number of phages have been reported infecting a variety of marine bacteria including RLOs (see detailed list in Friedman and Crosson, 2012). Hyperparasites have been shown to influence primary host–parasite relationships and recent renewed interest in phage therapy has highlighted the importance of the interplay among host immune response, primary pathogen and hyperparasite (Sabelis et al., 2002). Given the intracellular nature of the RLOs and their location in the digestive epithelium, the host immune response may play less of a role in WS than in bacterial septicemia. Infection of a prokaryote by a phage results in death of the host cell by lysis (lytic cycle; see reviews by Young, 1992, 2002) or by induced changes in physiology through co-opting of the host cell to produce more phages, and by lysogeny (Kourilsky, 1973; Weinbauer, 2004; Bobay et al., 2013). Lytic phages have been used as a therapeutic alternative to antibiotics in many systems including those of bacterial pathogens in marine species. Bacteriophages were recently reported as an alternative control for *Vibrio anguillarum* infecting Atlantic salmon (Higuera et al., 2013) and *V. parahaemolyticus* infections in brine shrimp (Martínez-Díaz and Hipólito-Morales, 2013). In both cases, efficacy of the phage therapy was based on phage-induced lysis of its bacterial host. Some phages integrate into the host genome (lysogenize) and are replicated and passed on to the next generation via host cell division (Kourilsky, 1973; Golais et al., 2013). Lysogeny has been shown to increase the virulence and pathogenicity of bacterial pathogens including those infecting marine species such as *V. harveyi* in shrimp (Ruangpan et al., 1999; Khemayan et al., 2006). Shrimp infected with phage-lysogenized *V. harveyi* experienced over 100 times the death rate of those lacking infection with the phage *V. harveyi* siphophage 1 (VHS1; Intaraprasong et al., 2009).

The phage infecting the WS-RLO appears unique in its ability to alter the course of infection, not by lysis of its bacterial host but apparently by effectively eliminating normal function of the WS-RLO. This is evidenced by a reduction in the effect of PE infection on the DG and resulting reduced mortality of the abalone host and functioning as a natural phage therapy. Approximately half of the WS-RLO colonies were phage infected (RLOv), thereby effectively reducing the pathogenic infection load by half. Increased host tolerance to WS-RLO infection in the PE may also contribute to the observed reduction in metaplasia and DG-RLO loads. Lysis of RLOv cells has not been observed by electron microscopic examination (Friedman and Crosson, 2012), but presumably occurs at some level in order to effect phage transmission. Alternatively, after its initial infection of the WS-RLO, transmission of the phage may rely primarily on lysis of the host abalone gastrointestinal epithelial cells by the RLOv followed by infection of new host cells and or hosts. Lysis of WS-RLO infected gastrointestinal cells has been observed by electron microscopy (Moore and Friedman, unpublished data). A lack of RLOv lysis suggests that the phage may be defective or that signals to induce host cell lysis are rare (Young, 1992, 2002; Golais et al., 2013).

## CONCLUDING REMARKS

It is encouraging that black abalone are surviving longer and recruiting to SNI. This does not seem to be fully explainable by stabilized SSTs in years since the original mass mortality. While our results suggest that some abalone at SNI have evolved increased resistance to the RLO, evidence for an evolved response was not strong. Differences in survival between exposure histories and tolerance to infection were enhanced with phage presence. The phage seen in our experiments substantially increased the longevity of infected abalone and, if prevalent in the wild, might help this endangered species recover from the brink of extinction.

## AUTHOR CONTRIBUTIONS

All authors helped write and edit the manuscript with Carolyn S. Friedman as the primary author. Carolyn S. Friedman also designed the experiments, read slides, analyzed data, made and or edited tables/figures. Kevin D. Lafferty analyzed data, created **Figure 2**, and designed **Figure 3**. Nathan Wight and Lisa M. Crosson conducted Trials 1 and 2, extracted DNA, sampled animals, and conducted qPCR assays. Glenn R. VanBlaricom collected abalone for experiments, conducted temperature analyses, and created **Table 2**.

## Conflict of Interest Statement

The authors declare that the research was conducted in the absence of any commercial or financial relationships that could be construed as a potential conflict of interest.
